# Chemopreventive and Anticancer Property of Selenoproteins in Obese Breast Cancer

**DOI:** 10.3389/fphar.2021.618172

**Published:** 2021-04-16

**Authors:** Supriya Bevinakoppamath, Adel Mohammed Saleh Ahmed, Shobha Chikkavaddaraguddi Ramachandra, Prashant Vishwanath, Akila Prashant

**Affiliations:** Center of Excellence in Molecular Biology and Regenerative Medicine, Department of Biochemistry, JSS Medical College, JSS Academy of Higher Education & Research, Mysore, India

**Keywords:** obesity, breast cancer, selenoprotein, organoselenium compound, oxidative stress

## Abstract

Obesity is a significant risk factor for various cancers including breast cancer resulting in an increased risk of recurrence as well as morbidity and mortality. Extensive studies on various pathways have been successful in establishing a biological relationship between obesity and breast cancer. The molecular classification of breast cancer includes five groups each having different responses to treatment. Increased levels of inflammatory cytokines seen in obese conditions drive the pro-proliferative pathways, such as the influx of macrophages, angiogenesis, and antiapoptotic pathways. Increased peripheral aromatization of androgens by aromatase increases the circulating estrogen levels which are also responsible for the association of obesity with breast cancer. Also, increased oxidative stress due to chronic low-grade inflammation in obese women plays an important role in carcinogenesis. Despite the availability of safe and effective treatment options for breast cancer, obese women are at increased risk of adverse outcomes including treatment-related toxicities. In the recent decade, selenium compounds have gained substantial interest as chemopreventive and anticancer agents. The chemical derivatives of selenium include inorganic and organic compounds that exhibit pro-oxidant properties and alter cellular redox homeostasis. They target more than one metabolic pathway by thiol modifications, induction of reactive oxygen species, and chromatin modifications to exert their chemopreventive and anticancer activities. The primary functional effectors of selenium that play a significant role in human homeostasis are selenoproteins like glutathione peroxidase, thioredoxin reductase, iodothyronine deiodinases, and selenoprotein P. Selenoproteins play a significant role in adipose tissue physiology by modulating preadipocyte proliferation and adipogenic differentiation. They correlate negatively with body mass index resulting in increased oxidative stress that may lead to carcinogenesis in obese individuals. Methylseleninic acid effectively suppresses aromatase activation thus reducing the estrogen levels and acting as a breast cancer chemopreventive agent. Adipose-derived inflammatory mediators influence the selenium metabolites and affect the proliferation and metastatic properties of cancer cells. Recently selenium nanoparticles have shown potent anticancer activity which may lead to a major breakthrough in the management of cancers caused due to multiple pathways. In this review, we discuss the possible role of selenoproteins as chemopreventive and an anticancer agent in obese breast cancer.

## Introduction

Obesity is a common disorder that not only leads to several health concerns like cardiovascular diseases, type 2 diabetes, and hypertension but also increases the risk for many types of cancer such as breast, colorectal, esophagus, and pancreas. Globally, more than 650 million adults are obese, above 1.9 million adults are overweight and about 2.8 million deaths have been reported as a result of being overweight or obese (Ahirwar and Mondal, 2019). The prevalence of overweight and obesity has increased from 8.4 to 15.5% and 2.2–5.1% respectively between the period of 1998 and 2015 in India. Luhar S et al. have stated that the prevalence of overweight and obesity will be around 30.5% (27.4–34.4%) and 9.5% (5.4–13.3%) among men, and 27.4% (24.5–30.6%) and 13.9% (10.1–16.9%) among women, respectively, by 2040 (Luhar et al., 2020). Studies have shown that approximately 20% of cancer cases are caused by excess weight and obesity which, in turn, may be due to a diet rich in fat and reduced physical activity (Wolin et al., 2010). This risk for pathological conditions starts from a body mass index (BMI) of around 21 kg/m^2^ (Pradeepa et al., 2015). Roberts et al. has emphasized that obesity is complex multisystem pathophysiology, and it is unlikely that there is a “one system fit all” mechanism (Roberts et al., 2010). The link between obesity and cancer risk has been demonstrated to be sex- and cancer-site-specific. Various biological mechanisms have been hypothesized to explain this link, a few of them being insulin and insulin-like growth factors, sex hormones, and adipokines. As a result of several shortfalls in these hypotheses, newer mechanisms like obesity-related hypoxia shared genetic susceptibility, and migrating adipose stromal cells have been proposed. Breast cancer is one of all the cancers whose risk increases with obesity.

The global increase in the incidence of breast cancer and rapidly increasing deaths related to cancer places breast cancer on the top of the chart (Fahad Ullah, 2019). The 2018 GLOBOCAN announced a total of 162,468 new cases of breast cancer and 87,090 deaths, representing it as the first-class cause of death among Indian women (Bray et al., 2018). In 2016, the Burden of breast cancer in respect of Disability-adjusted life-years (DALYs) per 100,000 females was 203.5 and it is expected to be 218.4 and 233 in 2021 and 2026, respectively. Years lived with disability (YLD) to Years of life lost (YLL) was estimated to increase from 64.1% in 2016 to 64.5 and 65.3 in 2021, 2026, respectively (Kunnavil et al., 2016). The mortality rate due to breast cancer is high in metropolitan cities of India (Malvia et al., 2017). This quantitative information provided indicates the exponential rise in cases of breast cancer, and there is an urgent need to probe into the possible causes and to plan for preventive interventions.

A combination of appropriate diet and physical activity can prevent nearly 40% of all cancers in a relatively new approach for the management of cancer, termed chemoprevention (Naithani, 2008). This involves ingesting dietary or pharmacological agents that are capable of modulating the process of carcinogenesis by preventing, delaying, or reversing cancer thus reducing cancer mortality (Naithani, 2008; Baliga et al., 2011). Selenium (Se) is an essential trace element and cofactor of various antioxidant enzymes that has emerged as a successful agent in the prevention of cancer (Naithani, 2008; Ghadi, 2009; Medina et al., 2001; Marshall et al., 2011; Cao et al., 2014; Cai et al., 2016). By incorporating itself into a family of proteins called selenoproteins in the form of selenocysteine (Sec), it plays an important biological role in living organisms. Se-containing compounds are promising molecules in cancer treatment because of their prooxidative stress-inducing property. Chemical derivatives of Se include organic and inorganic compounds. Organic selenium compounds (Table 1) have higher selectivity and effectiveness compared to inorganic selenium compounds and also exhibit fewer side effects (Valdiglesias et al., 2010; Brigelius-Flohé and Maiorino, 2013; Chen et al., 2013). It has been extensively studied that the organic selenium compounds, through different mechanisms act as chemopreventive and anticancer agents (Álvarez-Pérez et al., 2018). Naturally occurring organoselenium can be supplemented through diet and an appropriate dose of the trace element selenium has many health benefits. The deficiency of dietary selenium increases the risk of various diseases including cancer development (Lau et al., 2017). Several *in-vitro* studies (Unni et al., 2001; Husbeck et al., 2006; Lu et al., 2015), research on model organisms (Ip et al., 2000; Unni et al., 2004; Unni et al., 2005; Wang et al., 2009), and clinical (Brooks et al., 2001; Rejali et al., 2007; Steevens et al., 2010; Jaworska et al., 2013; Marshall et al., 2017) and epidemiological (Clark, 1996; Duffield-Lillico et al., 2002; Duffield-Lillico et al., 2003; Harris et al., 2012; Ou et al., 2012) research have accumulated scientific evidence to support the protective role of selenium in cancer chemoprevention. In this review, we present the accumulated data on the use of selenium compounds in the prevention of breast cancer among obese individuals.

**TABLE 1:** Organoselenium compounds and their role in preventing obesity-associated breast cancer.

SI No	Name of the compound	Structure of the compound	Models used	Role in breast cancer	References
1	Selenomethionine	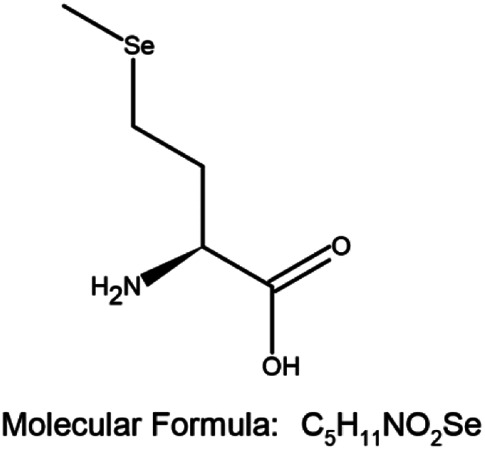	MCF-7 4T1.2 and female BALB/c mice	-Increases antioxidant enzymes and UCP2 protein expression, antioxidant damage reduction in lipid and proteins	Perou et al. (1999); Holliday and Speirs (2011); Chen et al. (2013)
				-Protection against breast cancer metastasis and tumor inhibition	
2	Selenocysteine	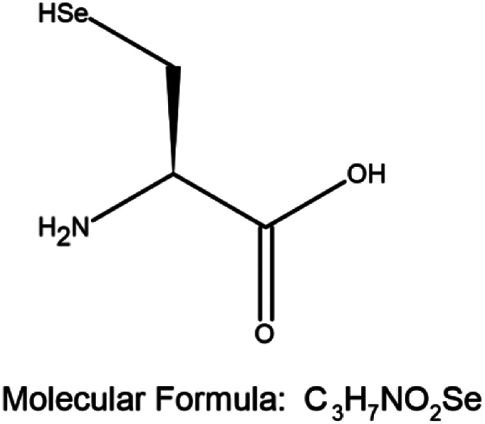	MDA-MB-231, MDA-MB-436, and MDA-MB-468	-S-phase cell cycle arrest	Fernandes and Gandin (2015)
				-Apoptosis	Long et al. (2015)
3	Methylselenocysteine	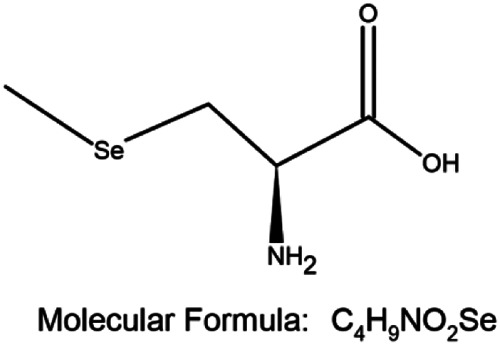	TM6	-Inhibits cell growth by inhibiting the activity of PI3-K and its downstream effector molecules in mouse mammary tumor cells *in vitro*	Unni et al. (2005)
4	Ebselen	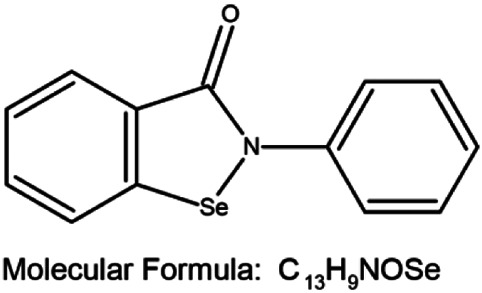	MCF-7, Zucker diabetic fatty (ZDF) rats	-Cancer prevention in combination with radiotherapy	Mahadevan et al. (2013); Thabet and Moustafa (2017); Dong et al. (2020)
				- Reduces the levels of fasting blood glucose	
5	Methyl seleninic acid	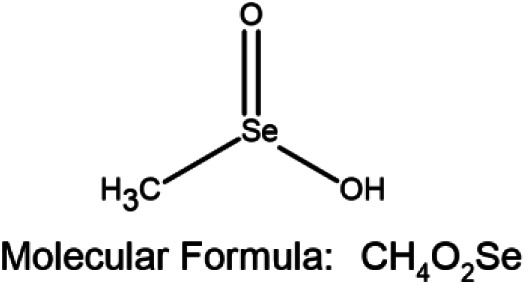	MDA-MB-231, MDA-MB-157, and BT-549	-Enhances the anticancer efficacy of paclitaxel in triple-negative breast cancer. Growth inhibition of triple-negative BC	Qi et al. (2012); Qiu et al. (2019); Sánchez-Jiménez et al. (2019); Tan et al. (2019)
			SCID mice bearing MDA-MB-231 triple-negative breast cancer xenografts	-Cytotoxicity	
			Mouse 4T1 cells	- Downregulation of PII and PIV promoters of CYP19A1 gene through the MAP kinase and JAK/STAT pathway induced by leptin/insulin ligand binding	

## Breast Cancer

The breast is constructed of two main forms of tissues i.e., stromal (supporting) tissues and glandular tissues. Glandular tissues include the milk-producing glands (lobules) and ducts (milk passages), while stromal tissues are made up of fibrous and fatty connective tissues of the breast. Cell fluids and waste are extracted by the breast lymphatic tissue immune system. Microarray-based gene expression profiling has revealed that breast cancers are intricate, and their histology is highly variable. Nevertheless, these studies have contributed their experimental results to classify tumors into different types based on the lymph node status, tumor grade, molecular type, and the presence of predictive markers such as estrogen receptor (ER), progesterone receptor (PR), and human epidermal growth factor receptor (HER2) (Perou et al., 1999; Holliday and Speirs, 2011). A prognostically important differentiation hierarchy occurs among all breast cancers and is classified into Luminal-A (ER^+^, PR^+/−^, HER2^−^), Luminal-B (ER^+^, PR^+/−^, HER2^+^), Basal/Triple-negative (ER^−^, PR^−^, HER2^−^), HER2 (ER^−^, PR^−^, HER2^+^) and Claudin-low (ER^−^, PR^−^, HER2^−^) which are also referred to as intrinsic subtypes (Kennecke et al., 2010). Cancer heterogeneity and diagnosis rely on the Union for International Cancer Control (UICC) and the American Joint Committee on Cancer (AJCC) TNM (Tumor Node Metastasis) staging system. The introduction of TNM classification has helped clinicians to better understand the progression of cancer and the treatment strategies along with further research on preventive strategies (O'Sullivan et al., 2017; Cserni et al., 2018). The extent of the disease severity depends upon the stages of cancer (Fouad et al., 2017). Invasive and noninvasive cancers are the two types of breast cancers depending upon the site of invasion. Most breast cancers begin in the cells that line the lobules and ducts and are referred to as ductal and lobular cancers respectively (Sharma et al., 2010). These cancer types can be diagnosed through mammography; however, this technique has some limitations when it comes to diagnosing women with high-density breast tissue (Nazari and Mukherjee, 2018).

Ductal carcinoma *in-situ* (DCIS) is the most common non-invasive breast cancer (Sharma et al., 2010) and acts as a precursor for invasive breast cancer. When epithelial breast cells undergo abnormal cell division and accumulate in their ducts and lobules it is referred to as DCIS (Allred, 2010; Gorringe and Fox, 2017). Age, ethnicity, tumor size, ER status are some of the reasons for the mortality due to DCIS (Narod et al., 2015). The strong correlation between obesity and DCIS has been demonstrated by recent research. Women with a BMI of ≥30 kg/m^2^ have shown a 1.6-fold rise in DCIS growth relative to any other second breast cancer during initial diagnosis (Flanagan et al., 2018). Lobular carcinoma *in situ* (LCIS) is non-invasive breast cancer and is considered as one of the markers to predict the increased breast cancer risk (Sharma et al., 2010). Postmenopausal women with ages ranging from 40 to 50 years are commonly diagnosed with LCIS. Unlike DCIS, LCIS is not an obligate precursor for breast cancer since the subsequent cancers occur in the contralateral breast (Flanagan et al., 2015). Immunohistochemical stains for LCIS and its variants have exhibited a complete or partial loss of cell-cell adhesion protein epithelial-Cadherin (E-cadherin) expression and the presence of p120 cytoplasmic catenin which is considered as the positive marker for LCIS (Chen et al., 2009; Wen and Brogi, 2018). Pleomorphic lobular carcinoma *in situ* (PLCIS) is a variant of LCIS and shows similar histopathological features as DCIS. PLCIS is further classified into 1) non-apocrine and 2) apocrine (Chen et al., 2009). Mammographic results have shown that the solid pattern of PLCIS shows significant Pleomorphic calcification (Downs-Kelly et al., 2011; Khoury et al., 2014; Pieri et al., 2014; De Brot et al., 2017). Although the BMI does not affect LSIC, breast density does. Breast density acts as an additional risk factor for LCIS growth (Minami et al., 2020). Infiltrating or Invasive Ductal carcinoma (IDC) - The propagation of IDC is only restricted to the milk ducts in the breast. Recent animal studies have demonstrated the invasive nature of cancer cells in absence of E-cadherin thus reducing the potential of metastasis (Padmanaban et al., 2019). BMI or obesity has no significant correlation suggesting there is no link between BMI and increased risk of IDC (Newcomb et al., 2011). Infiltrating lobular carcinoma (ILC) (invasive) ILCs are homogenous small cell tumors with low nuclear grade (Silverstein et al., 1994). Among all invasive breast cancers, ILC constitutes 2.3% and exhibits more HER-2 expression (Lee et al., 2010). Patients with ILC show low histological grade and lower prognosis compared to IDC (Metzger-Filho et al., 2019). Nulliparous women with a BMI ≥25 kg/m^2^ and women with a high BMI are at a higher risk of developing ILC (Newcomb et al., 2011).

## Impact of Obesity on Breast Cancer

Various factors have been investigated in several studies to explain the link between obesity and cancer risk. Histologically, adipose tissue constitutes 56% of non-lactating breast tissue and 35% of lactating breast tissue. Mammary adipose tissue is distinct from the subcutaneous adipose tissue found in other internal organs because it undergoes cyclic structural changes in response to the female hormone cycle and is in constant interaction with epithelial cells. During the development of the breast, epithelial cells penetrate the mammary fat pad producing branching ducts and terminal buds that create a fat-embedded glandular structure. In adult women, mammary glandular epithelial cells distinguish to a secretary and lactating alveoli, with simultaneous regression of mammary adipocytes under the hormonal influence through pregnancy or lactation, and the volume of glands and mammary adipocytes returns to the original conditions on cessation of these events. Breast tissue tumorigenesis recapitulates this natural physiological process, i.e. adipocyte differentiation, epithelial glandular cell proliferation, and extracellular matrix (ECM) remodeling. Reciprocal interactions between epithelial cells and adipocytes are often identified during the cancer process: tumor development, tumor growth, invasion, and metastases (Hovey and Aimo, 2010). Several overlapping pathways have been implicated in the increased risk of breast cancer in obese individuals. These include high circulating insulin/insulin-like growth factors-I (IGF-I), increased aromatase-mediated estrogen production, altered adipokine concentrations, and their signaling pathways promoting a chronic inflammatory condition.

Like leptin, insulin is also a major risk factor for the development of breast cancer in postmenopausal women as it induces the activity of adipose aromatase. In a more complex network between leptin and other factors, leptin and estrogen metabolism are shown to be related. Consequently, obesity-associated hyperleptinemia is suggested as a primary mediator for breast cancer pathophysiology (Howe et al., 2013; Crespi et al., 2016). Higher insulin and IGF-1 are capable of developing a pro-cancerous environment. Specifically, insulin or IGF-1 binding to their associated receptors activates a series of events including the phosphatidylinositol 3 kinase (PI3K)/Akt/mammalian target of rapamycin (mTOR) signal transduction cascade. This pathway leads up to the activation of S6 kinase and releasing the inhibition of the eukaryotic initiation factor-4e (eIF4e), a transcription factor. This cascade cumulatively contributes to the expression of cell cycle progression proteins (Arcidiacono et al., 2012; Crespi et al., 2016). It has been studied that in breast cancer, PI3K/AKT controls the leptin-mediated-epithelial mesenchymal transition. Additionally, the other research has recently suggested that leptin promotes breast cancer cell proliferation, migration, and invasion via the up-regulation of PI3K/AKT/SREBP2 signal pathway through acetyl-CoA acetyltransferase 2 (ACAT2) (Wei et al., 2016; Huang et al., 2017).

**FIGURE 1:**
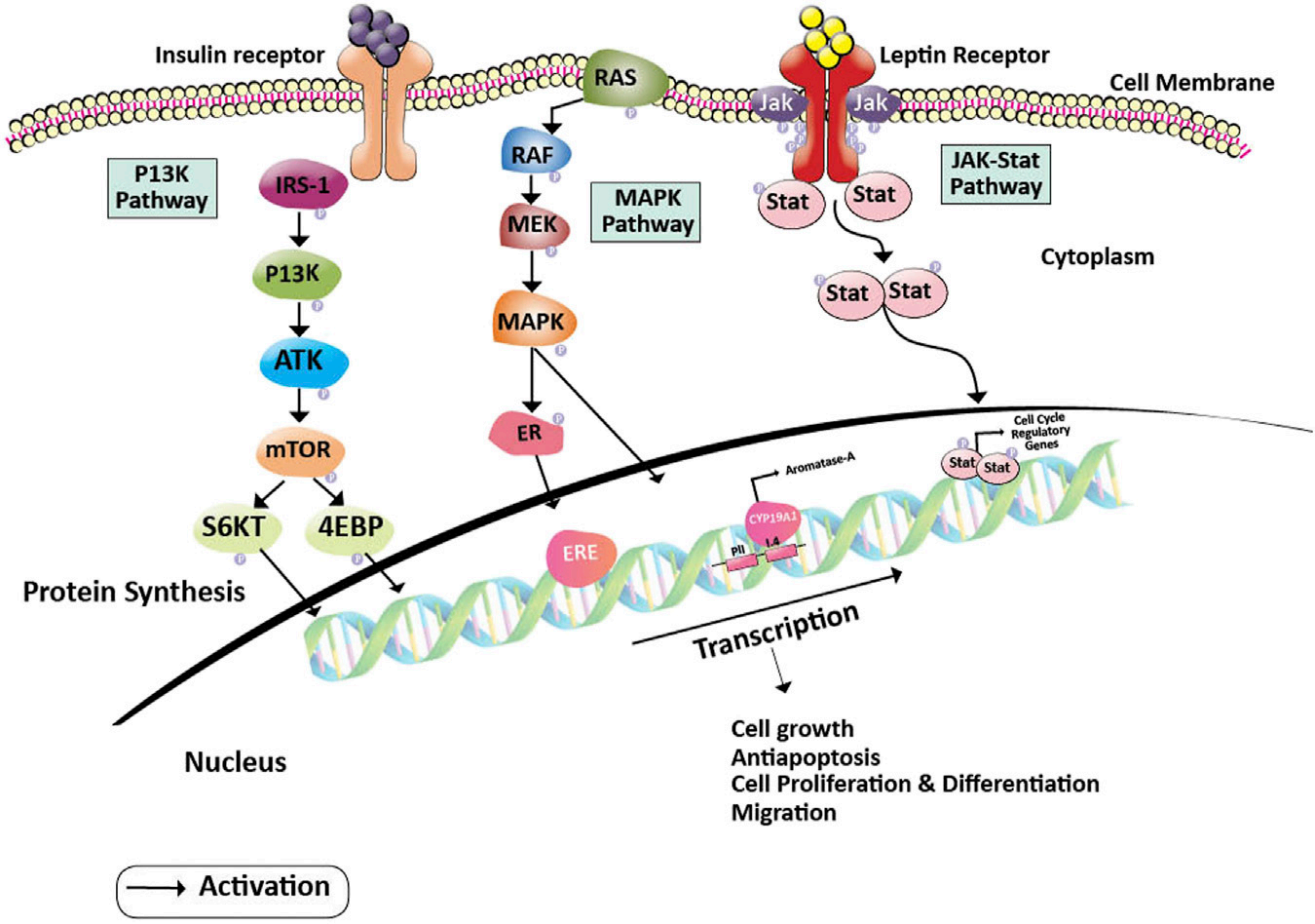
Pathway showing oncogenesis by the induction of Leptin and Insulin pathways. Insulin and Leptin binding to their receptors activates series of pathways including PI3K, MAPK, and JAK-Stat pathways, where, PI3K pathway leads to the activation of S6KT and releases the 4 EBP proteins, MAPK pathway gives rise to the activation of ERE and activation of aromatase gene and JAK-Stat dimerizations gives rise to the activation of cell regulatory elements respectively. Activation of these pathways helps in cancer cell growth, proliferation, differentiation, and migration. Abbreviations: 4EBP, Eukaryotic Translation initiation factor 4E binding protein; CYP19A2, Cytochrome P450 Aromatase; ER, Estrogen receptor protein; ERE, Estrogen responsive element; IRS-1, Insulin receptor substrate; JAK, Janus kinase; MAPK, Mitogen-activated protein kinase; MEK, Mitogen-activated protein kinase; mTOR, Mammalian target of rapamycin; PI3K, Phosphoinositide 3-kinase; RAF, Rapidly Accelerated Fibrosarcoma; RAS, Rat sarcoma; ROS, Reactive oxygen species; S6KT, Ribosomal protein S6 kinase beta-1; Stat, signal transducer and activator of transcription.

Leptin has also been shown to facilitate the growth and progression of neoplastic breast cancer cells by stimulating the pathway of Janus Kinase/Signal transducer and activator of transcription (JAK2/STAT3). Once leptin attaches to its receptor, the leptin receptor cytoplasmic domain recruits JAK2 kinase and phosphorylates the tyrosine residue. This results in the STAT3 protein anchorage, which is recruited from the SH2 domain. When STAT3 is bound to the receptor, the proteins JAK2 and STAT3 are phosphorylated to dimerize, allowing them to be translocated to the nucleus. They act as transcription activators for a variety of genes, such as c-myc, cyclin D1, p21/waf1, c-jun, junB, erg-1, and Bcl-2, all of which are involved in cell growth and proliferation (Banks et al., 2000). Insulin and leptin receptors activate the MAP kinase pathway leading to the activation of the estrogen receptor expression (Sánchez-Jiménez et al., 2019) (Figure 1).

Obesity being a state of chronic low-grade sterile inflammation presents with increased levels of circulating pro-inflammatory factors without clinical signs of inflammation (Mraz and Haluzik, 2014). Adipose tissue is involved in the regulation of physiological and pathological processes by releasing a variety of adipokines like leptin, adiponectin, and resistin, and other pro-inflammatory cytokines. Trace elements like selenium play an important role in the protection against inflammation, hence are the crucial factors required to suppress the complications related to obesity including carcinogenesis (Azab et al., 2014). Selenoproteins act by suppressing oxidative stress, regulating thyroid hormones, interrupting pancreatic insulin secretion, and regulating phosphorylation of energy metabolic pathways, like insulin signaling inhibitor protein tyrosine phosphatase 1B.

## Physiological Characteristics of Selenoproteins

Enzymes such as glutathione peroxidase (GPx), thioredoxin reductases (TR), iodothyronine deiodinases (DIO), selenoprotein *P* (SePP1) are the important selenoproteins present in our system.

### Glutathione Peroxidase

GPx is a family of closely related antioxidant enzymes that occurs in different forms i.e. GPx1 to GPx8. All the members of the family are selenium-containing proteins except GPx5. GPx1 detoxifies hydrogen peroxide and is expressed ubiquitously. GPx2, GPx3 modulate carcinogenesis and peroxidation in case of low glutathione respectively (Brigelius-Flohé and Maiorino, 2013). A cell could undergo apoptosis through various mechanisms and ferroptosis is one of them in which cell death is due to the production of iron-dependent reactive oxygen species (ROS) (Yang et al., 2014). A study conducted on human fibroblast cells elucidated that GPx4 being one of the homologs of GPxs, prevents cells from undergoing lipid peroxidation and regulates the ferroptosis cell death pathway. GPx4 also helps cells to avoid cell death induced by hydrogen peroxide by reducing the intracellular radical levels (Rohr-Udilova et al., 2018). In the absence of a reducing substrate, GPx7 stabilizes the oxidized peroxidase in its catalytically active form 1-Cys-GPx (Maiorino et al., 2015). *In-vitro* and *in-vivo* studies in recent years have come up with some interesting results where GPx7 acts as a catalyst for oxidative protein folding by utilizing Ero1-flavoproteins generated hydrogen peroxide (H_2_O_2_) suggesting GPx7 as a scavenger for hydrogen peroxide (Wang et al., 2014). GPx activity is shown to be significantly decreased in obese patients and there exists an inverse relationship between BMI and erythrocyte GPx activity (Amirkhizi et al., 2014). This increases the obesity-induced oxidative stress causing DNA damage leading to modified bases or mutations in tumor suppressor genes, a critical factor in carcinogenesis. This mounting evidence suggests that the GPx enzyme family has a significant role in carcinogenesis and also regulates the cancer stem cells and other signaling pathways making them ideal candidates for potential biomarkers or drug targets.

### Thioredoxin Reductases (TrxR)

TrxRs are flavoenzyme containing selenium and transfer reducing equivalents from NADPH to thioredoxin (Trx). They are the central enzymes in the Trx redox pathway. Trx acts as a reducing substrate in the reduction of ribonucleotides and detoxification of radicals and oxidants (Saccoccia et al., 2014)**.** TrxR family comprises three isozymes, TrxR1, TrxR2, and TrxR3 containing conserved Sec encoded by TGA which is vital for its catalytic activity. Each of these members is encoded by dinucleotide reductases namely TXNRD1, TXNRD2, and TXNRD3 respectively (Onodera et al., 2019). Sec is the direct target of reactive oxygen species (ROS), excessive production of which has been implicated in carcinogenesis. Also, due to the increased expression of TrxRs in many malignant cells, they are thought to be involved in different stages of carcinogenesis (Karlenius and Tonissen, 2010). Trx/TrxR system also plays a significant role in the physiology of the adipose tissue (Tinkov et al., 2018). Inhibition of intracellular signaling pathways downstream of insulin stimulation is responsible for TrxR1 induced suppression of anabolic metabolism and adipogenesis (Peng et al., 2016). Thioredoxin reductase inhibitors act as potential drugs in the treatment of cancer by blocking the transfer of reducing equivalents to antioxidant enzymes thus, generating oxidative stress or inhibiting the reduction of ribonucleotide thus decreasing the rate of DNA synthesis (Jia et al., 2019). Inhibition of TrxR reduces the reduced Trx thus increasing the oxidized Trx in the cells which binds to a number of apoptosis regulating proteins thus promoting apoptosis. Hence, targeting TrxR/Trx for inhibition has been developed as a potential therapeutic option for combating obese cancers with several natural inhibitor molecules being identified e.g., curcumin, alantolactone, etc.

### Iodothyronine Deiodinases

DIO is a family of selenoenzymes that consists of three members, DIO1, DIO2, DIO3, and share catalytic and sequences homology (Bianco et al., 2002). DIOs are not much involved in oxidative stress regulation as other selenoproteins. However, DIOs are more active in the thyroid system where they convert T4 (thyroxin) to T3 (triiodothyronin). DIO2 is highly expressed and plays an important role in brown adipose tissue function. The deiodinase enzyme levels may be upregulated or downregulated in the tumor cells depending on the status of the critical intracellular signaling pathways, such as Wingless-related integration (Wnt), mitogen-activated protein kinase (MAPK), and Sonic-hedgehog (Shh) thus reducing its catalytic efficiency (Goemann et al., 2018). A variety of clinical conditions may be addressed by manipulating the intracellular T3 concentrations by pharmacological agents that act through deiodinase modification. Recent studies have shown that increased expression of DIO3 enhances tumor proliferation suggesting the possible roles of deiodinases as cancer makers and potential modulators of tumor progression (Goemann et al., 2017).

### Selenoprotein P

Sepp1 is a secreted glycoprotein found in plasma containing multiple Sec residues and thought to have antioxidant properties. Selenium is regulated in the liver and is transferred to the organs utilizing Sepp1 which serves as a measure of selenium intake status (Hill et al., 2012). The selenium content of the whole body is controlled by the excretion of Se from the liver. Sepp1 significantly responds to proinflammatory stimuli involved in the pathogenesis of obesity and associated metabolic disturbances. Polymorphisms in selenoproteins are associated with variations in protein levels and pose a cancer risk to the system. These types of polymorphisms are seen in the 3’UTR of mRNA (Kadkol and Diamond, 2020). Studies have also reported an inverse relationship between cancer risk and Sepp1 levels (Cui et al., 2017).

## Selenoproteins in the Pathogenesis of Obesity

The selenoproteins are expressed in adipose tissue of both healthy and obese individuals indicating the significant roles of selenium in adipocyte biology (Zhao et al., 2015). Inside the cells, selenocompounds are metabolized into selenide to become bioavailable and will be utilized to produce selenocysteine. Selenocysteine lyase (SCLY) uses the Sec from dietary sources, degradation of selenoproteins, and also from the metabolism of selenomethionine (SeMet). The enzyme SCLY decomposes Sec into alanine and selenide. As the free Sec is not present in the cells, this process of recycling the selenium may act as a source of this micronutrient. Targeted removal of the tRNA ([Ser]Sec) gene (Trsp) in mice revealed an increase in apolipoprotein E (ApoE) that was accompanied by elevated plasma cholesterol levels and upregulation of genes involved in cholesterol biosynthesis, metabolism, and transport leading to increased cholesterol synthesis. Indicating that selenoproteins play a significant role in regulating lipoprotein biosynthesis and metabolism (Sengupta et al., 2008). High-fat diet-induced obesity changes the selenogenome expression by up-regulating 12 selenoprotein genes, down-regulating 13 selenoprotein genes associated with thioredoxin and oxidoreductase systems (Zhao et al., 2015). By the differential regulation of the gene expression for fatty acid *ß*-oxidation, Se suppresses the formation of fatty liver and induces inhibition of adipocyte hypertrophy and abdominal fat accumulation (Kim J. E. et al., 2012). Selenate exhibits an anti-adipogenic activity by activating the transforming growth factor-β1 (TGF-β1) receptor in the preadipocytes thus modulating adipogenesis which may be inhibited by a specific inhibitor of the TGF-β1 receptor (Kim C. Y. et al., 2012). Few other studies have also shown an inverse relation of selenium with obesity (Cui et al., 2017; Zhong et al., 2018). There was a significant reduction in the levels of thyroid-stimulating hormones (TSH), BMI, waist circumference, and percentage of visceral fat in obese patients with subclinical hypothyroidism and type 2 diabetes upon supplementation with L-selenomethionine 83 µg/day when compared to the control group. This shows that selenium modulates thyroid hormone synthesis and there exists a strong association between obesity and low selenium intake (Guarino et al., 2018). Hence it can be stated that selenium and its related compounds play a vital role in normal physiology, obesity, and its related complications.

## Organoselenium Compounds as Chemopreventive Agents in Obese Breast Cancer

Understanding the metabolism of organoselenium compounds is essential to determine whether the parent compound and/or its metabolites are responsible for chemoprevention. Methylselenocysteine is considered more efficacious when compared to all the other naturally occurring organoselenium compounds. With structural modifications, synthetic organic compounds can be tailored to achieve greater efficacy with minimal side effects (El-Bayoumy and Sinha, 2004). Upon being incorporated into selenoproteins in the cells and tissues, they participate in a series of homeostatic processes due to their capability to react with molecular oxygen and thiols. They exhibit antioxidant and detoxification functions and protect against a range of cellular and extracellular prooxidants (Bartolini et al., 2017). They prevent oncogene activation, maintain the redox tone of the tissues, and induce the expression of genes responsible for the early phase of the immune surveillance of tumor cells. Natural killer (NK) cell activity is increased by higher levels of selenium. Methylselenol, a metabolite of selenium, upregulates natural killer group 2 member D (NKG2D) ligands on the surface of the tumor cells which are then recognized and eliminated by the CD8^+^ T cells also expressing NKG2D (Hagemann-Jensen et al., 2014). Studies are demonstrating lower NK cell numbers in the blood and reduced interaction of NK cell-tumor cells in the lungs of diet-induced obese mice injected with breast cancer cells when compared to lean animals (Spielmann et al., 2017). This indicates that impaired NK cell function could be a possible mechanism for increased cancer risk observed during obesity. Hence, it may be hypothesized that improving the NK cell function with selenium supplementation in obese individuals may reduce their risk of cancer development. However, large-scale clinical trials are required to further ascertain the fact that may prove beneficial to a larger population. Major clinical research and epidemiological studies that are conducted to date have been summarized in Table 2.

**TABLE 2:** Major clinical research and epidemiological studies conducted to date.

SI No	Population	Number of subjects	*Cancer* research	Conclusion of the study	References
1	Malaysia	62 (cases and control each)	Breast cancer	Breast cancer is associated with low serum selenium levels	Rejali et al. (2007)
2	Poland	95 lung cancer, 113 laryngeal cancer, and corresponding controls	Lung and laryngeal cancers	Selenium level below 60 μg/L is associated with lung and laryngeal cancer	Jaworska et al. (2013)
3	Baltimore longitudinal study	52 (cases) and 96 (controls)	Prostate cancer	The risk of prostate cancer is high in low plasma selenium cases	Brooks et al. (2001)
4	The university of California, san francisco, California	568 with non-metastatic prostate cancer	Prostate cancer	Genetic variation in selenoproteins may be associated with high-grade disease and recurrence of prostate cancer	Gerstenberger et al. (2015)
5	Netherland	64 esophageal squamous cell carcinoma, 112 esophageal adenocarcinomas, and 114 gastric cardia adenocarcinoma cases	Esophageal and gastric cancer	There is an inverse association between toenail selenium and risk of esophageal squamous cell carcinoma, esophageal adenocarcinoma, and gastric cardia adenocarcinoma	Steevens et al. (2010)
6	University of North Carolina hospital, North Carolina	133 cases	Colorectal adenoma	The prevalence of colorectal adenoma is less with high selenium levels	Connelly Frost et al. (2006)
7	Swedish	3,146 cases	Breast cancer	The survival rate is higher among the subjects who take selenium before the diagnosis of breast cancer	Harris et al. (2012)
8	Eastern United States of America	1,312 cases	Prostate cancer	Selenium reduces prostate cancer incidence	Duffield-Lillico et al. (2003)
9	Szczecin, Poland	546 cases	Breast cancer	Selenium level greater than 64.4 µg//L may be beneficial for breast cancer outcome	Lubinski et al. (2018)
10	Poland	97 (cases)	Women with any cancer	The serum level of selenium level should be maintained between 70 and 90 μg/L for protection against cancer	Narod et al. (2019)
		184 (control)			

**FIGURE 2:**
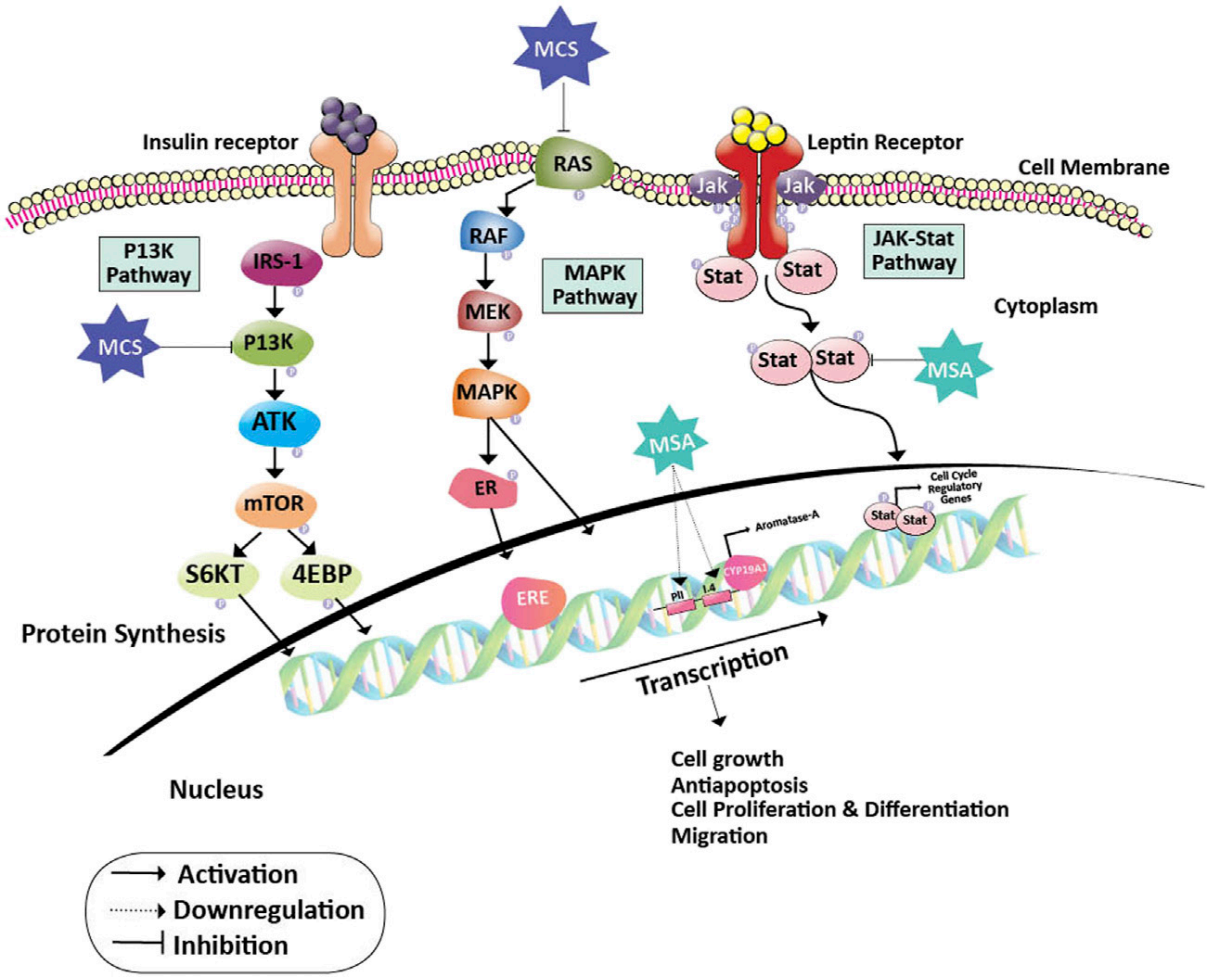
Action of Methylseleninic acid and Selenocysteine on oncogenic pathways. Inhibition of PI3K and RAS proteins by MCS halts the activation of transcription factors (S6KT and 4EBP) and MAPK pathway which is essential for the activation of cell cycle regulatory genes. Whereas, MSA down-regulates the PII and I.4 promoters of the aromatase gene leading to the formation of inadequate levels of estrogen. The action of MCS and MSA on their respective targets helps in the prevention of cancer cell growth, proliferation, differentiation, migration, and induces apoptosis. Abbreviations: 4EBP, Eukaryotic Translation initiation factor 4E binding protein; CYP19A2, Cytochrome P450 Aromatase; ER, Estrogen receptor protein; ERE, Estrogen responsive element; IRS-1, Insulin receptor substrate; JAK, Janus kinase; MAPK, Mitogen-activated protein kinase; MCS, Methylselenocysteine; MEK, Mitogen-activated protein kinase; mTOR, Mammalian target of rapamycin; MSA, Methylseleninic acid; PI3K: Phosphoinositide 3-kinase; RAF, Rapidly Accelerated Fibrosarcoma; RAS, Rat sarcoma; ROS, Reactive oxygen species; S6KT, Ribosomal protein S6 kinase beta-1; Stat, signal transducer and activator of transcription.

Estrogen and its metabolites play a pivotal role in the proliferation of hormone-sensitive breast cancer. Cytochrome P450 aromatase (CYP19) converts androgens to estrogens and is a key enzyme for the biosynthesis of estrogen. Hence, aromatase is an important target for breast cancer prevention and therapy as estrogen plays a critical role in obese postmenopausal breast cancer progression. Nevertheless, treatment with aromatase inhibitors is associated with several adverse effects like osteoporosis, cardiovascular, and neurocognitive defects due to systemic suppression of estrogen biosynthesis (Buzdar et al., 2006; Coombes et al., 2007). Methylseleninic acid (MSA) and Methylselenocystein (MCS) are two organoselenium derivatives that are more pronounced in the treatment of breast cancer. They function on cancer cells by blocking certain JAK/STAT, MAPK, and PI3K pathways that are important for cancer cell formation, proliferation, differentiation, invasion, and anti-apoptotic activity. In specific, MSA inhibits the STAT dimer and the PII and PI.4 promoters of the CYP19A1 gene that transforms androgens to estrogens. This inhibition of MSA contributes to the inactivation of regulatory genes and estrogen receptors in the cell cycle, eventually leading to the inhibition of cancer cell progression (Sánchez-Jiménez et al., 2019). In contrast, MCS operates on cancer cells by inhibiting the PI3K pathway which is either activated by binding leptin or insulin to their respective receptors. This inhibition halts the activation of S6 kinase and 4EBP. Also, MCS acts upon ER receptor and CYP19A1 expression by inhibiting the MAP kinase pathway. Thus, contributing to the prevention of cancer cell growth and differentiation (Unni et al., 2005) (Figure 2). It was later shown that cysteine sulfhydryl residues present in the catalytic domain of protein kinase C (PKC) undergo redox modification by the MSA-methylselenol redox cycle resulting in the inactivation of PKC by lower concentrations of MSA. Increased inactivation of the promitogenic and prosurvival PKC epsilon isoenzyme resulted in reduced cell growth and increased apoptosis (Gundimeda et al., 2008) and has also been shown to enhance insulin sensitivity. These studies have been performed on the *in-vitro* models of prostate cancer; however, similar principles may be applied to breast cancer as both organs are dependent on gonadal steroids for their development, tumors arising from them are hormone-dependent and have similar underlying biological processes.

Triple-negative breast cancer (TNBC) is an aggressive type of breast cancer that does not respond well to hormone therapies. Treatment options for TNBC are very limited and immediate needs for alternative therapies are indeed in demand. There are several mechanisms linking obesity and TNBC, including the role of insulin on the AKT/mammalian target of rapamycin (mTOR) signaling network, tissue inflammation, and protumorigenic tissue microenvironment driving the aggressive TNBC (Dietze et al., 2018). Selenium-containing polysaccharide extracted from *Pyracantha fortuneana* has shown to have anticancer activity against TNBC MDA-MB-231 cells by arresting the cells in the G2 phase and inhibiting CDC25C-CyclinB1/CDC2 pathway (Yuan et al., 2016). This pathway is shown to be hyperactive in obese (*ob/ob*) mice wherein complex-I is identified as a critical modulator of obesity-induced metabolic remodeling in *ß*-cells which in turn is regulated by cyclin-dependent kinase-1 (CDK-1) (Gregg et al., 2019). It has been shown that toxicity induced by selenium is effective in treating TNBC. Organic selenocyanate, when conjugated with bevacizumab and trastuzumab, decreased cell viability, and cell proliferation by generating superoxide anions in the presence of glutathione in TNBC cell lines MDA-MB-468 and MDA-MB-231 with less effect on normal cells (Khandelwal et al., 2018). Selenofolate, a selenoconjugate resulting from the conjugation of redox Se with folic acid and selenite has an effective cytotoxic effect on TNBC cell lines *in-vitro* (Khandelwal et al., 2020). The attachment of redox selenium to monoclonal antibodies serves as a carrier for selenium, targeting it to the TBNC cells. In the presence of obesity, whether there is excess production of superoxide anions by the organoselenium compounds in cancer as well as normal cells needs to be explored further.

By oxidative inactivation of the focal activating kinase (FAK kinase), GPx1 decreases the cell adhesion and proliferation as well as decreases the metastasis of TNBC cells to the lungs (Lee et al., 2020). A recent study shows that selenophosphate synthetase 2 (Sephs2), which is co-translationally incorporated into selenoproteins, is overexpressed in TNBC cells, providing new insight into the use of Sephs2 as a therapeutic target for treatment, because tumor aggressiveness can be associated with mRNA and protein concentrations (Nunziata et al., 2019)**.** GPx3 has been identified as a regulator of insulin receptor (IR) expression and insulin sensitivity in adipose tissue (Hauffe et al., 2020). Downregulation of IR is shown to be accompanied by the reduced mRNA expression of selenoproteins Txnrd3, Sephs2, and Gpx3. Adipose tissue of the obese patients shows reduced Gpx3 expression which may be induced by selenite treatment to enhance the IR expression. Contrasting expression patterns of Sephs2 proteins in TNBC and adipose cells may be investigated further to understand its exact role which may also be dependent on the chemical nature and the dosage of selenium used.

## Metastasis in Obese Breast Cancer and the Role of Selenoproteins

The mortality and morbidity seen in cancer patients are more due to the widespread metastasis rather than the primary tumors. Among the predictors of prognosis in metastatic breast cancer, obesity is an independent predictor of poor survival (von Drygalski et al., 2011). To evaluate the association of obesity and metastasis, progression of the tumor to metastasis of Py230 and EO771 grafts were studied by Bousquenaud M et al., who showed that tumors in obese mice grew faster, was less vascularized, more hypoxic, enriched with CD11b^+^Ly6G^+^ neutrophils in an ovariectomized C57BL/6 J female mice. Hence, they concluded that the incidence of metastasis and a higher ratio of triple-negative breast cancer is seen in obesity (Bousquenaud et al., 2018). In a retrospective study to compare the metastasis between obese and non-obese breast cancer women, it was shown that obesity is linked with visceral metastases development, especially lung and liver metastases (Osman and Hennessy, 2015).

Metastasis occurs by a different process like a local invasion, intravasation, and extravasation (Marciel and Hoffmann, 2017). The cancerous cell has the property of cell invasion by amoeboid cell movement which is acquired by inhibition of β1-integrin leading to collective to amoeboid transition (CAT) or by inhibition of proteases leading to mesenchymal to amoeboid transition (MAT) (Marciel and Hoffmann, 2017). This switching from MAT to CAT and vice versa can occur under certain conditions as in breast cancer metastasis (Melzer et al., 2017). The tight junction is an adhesive complex, keeps the cells together and maintains the cell integrity. The barrier formed by the tight junction should be altered by the tumor cell to penetrate and interact with vascular endothelium to metastasize. This barrier may be strengthened by selenium. A study by Martin et al. showed that selenium-treated MDA-MB-231 breast cancer cells have very less cell motility, reduced penetrance to endothelial cell layers and also it stabilizes the structure of the cell (Martin et al., 2007). Obesity may have profound effects on the extracellular matrix composition promoting local invasion and metastasis. Collagen VI which is upregulated in the extracellular matrix of obese tumor-bearing mice causes adhesion, migration, and invasion of human breast cancer cell lines through adhesion receptor, neuron-glial antigen 2 (NG2), and tyrosine kinase epidermal growth factor receptor (EGFR) receptor crosstalk, and Mitogen-activated protein kinases (MAPK) signaling activation (Wishart et al., 2020).

Intravasation is the local invasion of the cancerous cells into the vessel wall and thus transforming into circulating tumor cells. The mesenchymal and amoeboid cell invasion, neoangiogenesis, and vascular remodeling play a major role in intravasation. Other mechanisms that play a role are: 1) intravasation of macrophages via the epidermal growth factor or colony-stimulating factor 1, 2) signaling pathway of paracrine, 3) the microenvironment of the tumor, 4) the vessel wall elements surrounding the intravasation of tumor cell clusters, 5) cooperative intravasation and 6) intravasation associated with the vasculogenic mimicry (Marciel and Hoffmann, 2017; Zavyalova et al., 2019). In 40% of breast cancer, there is an expression of cyclooxygenase-2 (COX-2) which causes metastasis mediated by prostaglandins (Singh et al., 2007). Obese breast cancer patients have a worse outcome and do not respond well to aromatase inhibitor treatment and chemotherapy which may be linked to elevated COX-2 expression and the prostaglandin E2 (PGE2) (Subbaramaiah et al., 2012; Bowers and DeGraffenried, 2015). Selenium-activated AMP-activated protein kinase decreases COX-2 expressions via a COX-2/prostaglandin E (2) signaling pathway and hence mediates anticancer activity (Hwang et al., 2006; Cominetti et al., 2019).

The selenoproteins show a pivotal role as an anti-metastatic drug by involving in redox reactions, homeostasis of calcium, and mitigation of stress (Reeves and Hoffmann, 2009; Marciel and Hoffmann, 2017). In a study with dietary Se like sodium selenite, methylseleninic acid (MSA), or SeMet, a Se-deficient and a Se-adequate diet were fed to mice before mammary gland inoculation of 4T1.2 cells. It was seen that there was no significant difference in tumor growth and metastasis between the Se-adequate and Se-deficient groups but, the SeMet supplemented mice showed protection against breast cancer metastasis than selenite and MSA supplementations. They also found that selenite supplementation aggravated the metastasis incidence (Chen et al., 2013).

Oral administration of *Lactobacillus bravis* enriched with selenium nanoparticles (SeNPs) increased the lifespan of tumor-bearing BALB/c mice by a significant elevation in serum IL-17 and interferon γ (IFN-γ) and natural killer (NK) cell activity and also reduces liver metastasis (Yazdi et al., 2013). Nevertheless, IFN- γ, IL-2, IL-12, and TGF-β increase by supplementation of SeNPs as a vaccine helping to minimize tumor volume by activating an immune response (Yazdi et al., 2015). Hence, it can be stated that Se plays a pivotal role in chemoprevention and protect against cancer metastasis. Even though Se plays a major role in cancer metastasis prevention, the exact mechanisms of different forms of selenium compounds in cancer metastasis are yet to be identified. Thus, by understanding the exact mechanism of action of various selenocomponds, anti-metastatic therapies can be developed to reduce and treat cancer.

## Discussion

SeMet and Sec play a crucial role in redox state regulation in breast cancer cells. Particularly SeMet increases the redox status of cancer cells by increasing the expression of uncoupling protein 2 (UCP2) and redox enzymes and also reducing oxidative damage to proteins and lipids. In contrast to this, selenocysteine decreased the antioxidant enzyme and UCP2 protein expression, thereby increasing the ROS production and decreasing the viability of breast cancer cells (Pons et al., 2020). Upregulation of UCP2 in cancer leads to chemoresistance by decreasing ROS production. However, there is a continuous debate on the fact of whether UCP2 upregulation is a cause or effect of oxidative stress. Nonetheless, some studies have reported that UCP2 expression confers a pro-survival advantage for cancer cells (Pons et al., 2015). UCP2 is a mitochondrial anion that is upregulated in macrophages and human adipose tissue and has recently gained popularity in the field of obesity research. The metabolic activation of the macrophages in the obese adipose tissue may be responsible for the altered immune cell functioning which may be hypothesized to be mediated by UCP2. Hence, it would be interesting to study the effect of organoselenium compounds on the redox status of the cancer cells in the obese microenvironment and understand the cross-talks mediated by UCP2.

The damaged and dysfunctional intracellular components are delivered to the lysosomes for degradation by a process that is referred to as autophagy which is regulated by mTOR signaling. Compounds that induce or inhibit autophagy may be exploited for therapeutic applications against cancer. Seleno-purine (SLNN-15) is shown to stimulate anti-proliferative activity in MDA-MB-231 cell lines via inducing autophagy by selectively inhibiting the AKT-mTOR pathway (Chang et al., 2019). Upregulation of autophagy has been documented in several other chronic conditions, including obesity wherein inhibition of autophagy can protect the cells from obesity and insulin resistance (Jacob et al., 2017). Hence, it is still not clear whether autophagy is required for cell survival or cell death and it may be worthwhile to explore the role of organoselenium compounds in inducing or inhibiting autophagy in obese breast cancers.

**FIGURE 3:**
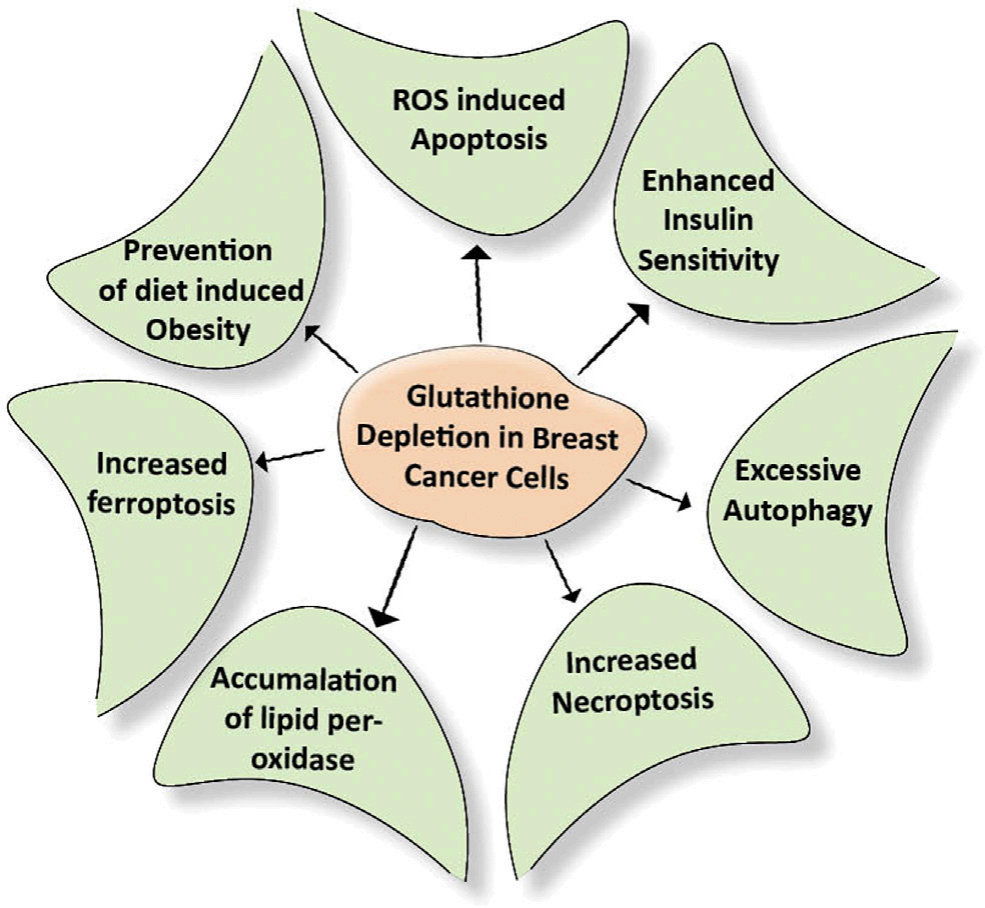
Effect of depletion of glutathione on cellular events. Breast cancer cells develop their antioxidant system under stress conditions. This system protects the cells from getting damaged due to external stimuli. Depletion of the glutathione damages the above-mentioned cellular events required for nourishing the cancer cells.

**FIGURE 4:**
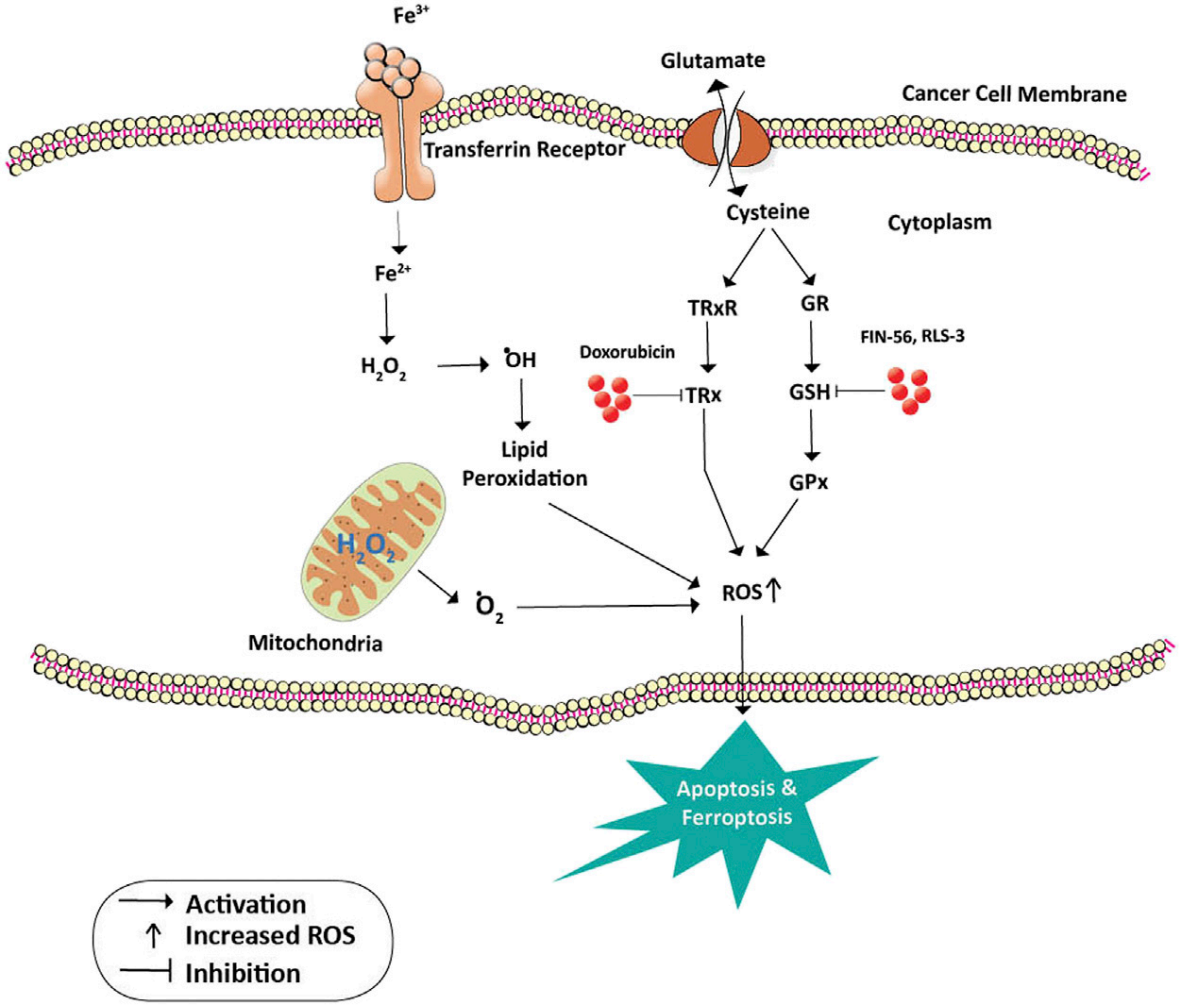
Inhibition of GPx and TRx in cancer prevention. Cysteine is a building block for the formation of GSH and TRx. GSH is a cofactor and substrate for GPx and is required for the lipid repair function of this enzyme. Loss of GSH enzyme due to the action of FIN-56 and RLS3 leads to the accumulation of unrepaired lipid peroxides and death of cancer cells by Ferroptosis. On the other hand, doxorubicin inhibits the action of TRx thus increasing the ROS levels and death by apoptosis. Oxidative stress on the mitochondria releases oxygen free radicals which also lead to apoptosis. Abbreviations: ˙O_2_, Oxygen free radical; ˙OH, Hydroxyl radical; Fe ^3+^ and Fe^2+^, Ferric and Ferrous ions; GR, Glutathione reductase; GSH, Glutathione; GPx, Glutathione peroxidase; H_2_O_2_, hydrogen peroxide; TRx, Thioredoxin; TRxR, Thioredoxin reductase.

Many cancer trials indicate that glutathione is of major significance in breast cancer. It is established that Glutathione depletion makes cancer cells vulnerable to chemotherapeutic drugs. Other effects of glutathione depletion are shown in Figure 3. Coupling of γ-glutamylcysteine by the catalysis of glutamine cysteine ligase (GSL) followed by the coupling of γ-glutamylcysteine with glycine catalyzed by glutathione synthase (GS) produce GSH and GSSH. When oxidative stress is induced in the mitochondria of cancer cells, the GSH is produced in the cytoplasm and prevents the cells from undergoing any damage. The redox status of GSH/GSSG is a significant predictor of cancer cell apoptosis. The decline in the GSH/GSSG ratio is consistently correlated with apoptosis. The decrease in GSH impairs the antioxidant system and increases the ROS generation, which speeds up mitochondrial damage and causes apoptosis (Zhao et al., 2012) (Figure 4). Necroptosis is an alternative type of programmed cell death with distinct characteristics in the cellular components. GSH depletion induces oxidative stress-induced necroptosis owing to pharmacological inhibition (Nagai et al., 2002; Galluzzi and Kroemer, 2008). RSL3 and FIN-56 have been shown to be an inducer of ferroptosis by covalently attacking the active GPX4 selenocysteine site and resulting in the aggregation of lipid ROS. However, the mechanism of RSL3-induced ferroptosis is not by depleting GSH but by inactivating GPX4. GPX4 silencing sensitizes RSL3-induced ferroptosis cells that are followed by lipid ROS accumulation (Yang et al., 2014; Feng and Stockwell, 2018). Thus, decreased GSH at the cellular level is shown to lead to apoptosis, necroptosis, and ferroptosis and affects the autophagic process. Depletion of glutathione has also been shown to combat diet-induced obesity. Overall, the potential association between GSH and autophagy in obese breast cancer also needs further study.

## Conclusion

Selenoproteins are potent modifiers of carcinogenesis and tumor progression. They inhibit tumor development by revoking the oxidative stress insults particularly in cancers that are driven by inflammatory mediators. Breast cancer and obesity both are chronic inflammatory conditions that present with increased oxidative stress and altered redox homeostasis. Decreased selenoproteins in adipose tissue may result in adipocyte dysfunction leading to insulin resistance, inflammation that may progress to breast cancer. Selenium supplementation may help in mitigating oxidative stress thus lowering the occurrence of carcinoma. However, this has been an area of debate as selenium supplementation without assessing the previous selenium status in individuals might be hazardous. Also, further studies have to be undertaken to precisely understand the biological mechanism of how selenium influences the tumorigenesis in obese breast cancer that belong to different molecular subtypes.
